# Multiple bacterial infections increase the risk of hepatic encephalopathy in patients with cirrhosis

**DOI:** 10.1371/journal.pone.0197127

**Published:** 2018-05-10

**Authors:** Lan-Ting Yuan, Seng-Kee Chuah, Shih-Cheng Yang, Chih-Ming Liang, Cheng-Kun Wu, Wei-Chen Tai, Tsung-Hsing Hung, Seng-Howe Nguang, Jiunn-Wei Wang, Kuo-Lun Tseng, Ming-Kun Ku, Pin-I Hsu, Deng-Chyang Wu, Chien-Ning Hsu

**Affiliations:** 1 Division of Gastroenterology, Yuan General Hospital, Kaohsiung, Taiwan; 2 Division of Hepato-gastroenterology, Department of Internal Medicine, Kaohsiung Chang Gung Memorial Hospital, Kaohsiung, Taiwan; 3 Chang Gung University, College of Medicine, Kaohsiung, Taiwan; 4 Division of Hepato-gastroenterology, Department of Internal Medicine, Buddist Tzu Chi General Hospital, Dalin Branch, Chia-Yi, Taiwan; 5 Division of Gastroenterology, Pin-Tung Christian Hospital, Pin-Tung, Taiwan; 6 Division of Gastroenterology, Department of Internal Medicine, Kaohsiung Medical University Hospital and Kaohsiung Medical University, Kaohsiung, Taiwan; 7 Division of Gastroenterology, Kaohsiung Municipal TaTung Hospital, Kaohsiung, Taiwan; 8 Division of Gastroenterology, Cishan Hospital, Kaohsiung, Taiwan; 9 Division of Gastroenterology, FooYin University Hospital, Pin-Tung, Taiwan; 10 Division of Gastroenterology, Department of Internal Medicine, Kaohsiung Veterans General Hospital, National Yang-Ming University, Kaohsiung, Taiwan; 11 Department of Pharmacy, Kaohsiung Chang Gung Memorial Hospital, Kaohsiung, Taiwan; 12 School of Pharmacy, Kaohsiung Medical University, Kaohsiung, Taiwan; Texas A&M University, UNITED STATES

## Abstract

**Objective:**

Patients with liver cirrhosis (LC) are at increased risk for bacterial infections. It is not fully understood how exposure to infections induces further development of hepatic encephalopathy (HE). This study estimated risks of infection associated with HE among patients with LC.

**Methods:**

A nested case-control study of 14,428 adult patients with LC was performed using the population-based Longitudinal Health Insurance Database 2000 in Taiwan. Cases were cirrhotic patients who developed HE during follow-up. Controls were matched to each case by age at LC diagnosis (±2 years), sex, Charlson Comorbid index score, year of LC, and follow-up time with a 1:1 ratio. A multivariate logistic regression model was used to determine and compare the odds of developing HE based on exposure to various risk factors, including site of infection, cirrhosis-related complications, *Helicobacter pylori* eradication therapy, and peptic ulcer bleeding. Patient survival was evaluated using the time-dependent Cox regression model.

**Results:**

Cirrhotic patients with HE (n = 714) and without HE (n = 714) were matched to compare risks. Infections and more frequent yearly infections were significantly associated with increased risk of HE. Independent predictors of HE included spontaneous bacterial peritonitis (aOR, 5.13; 95% CI, 3.03–8.69), sepsis (aOR, 2.54; 95% CI, 1.82–-3.53), and biliary tract infection (aOR, 2.03; 95% CI, 1.2–3.46), controlling for confounders.

**Conclusion:**

Frequent infections are associated with increased risk of HE in cirrhotic patients. More frequent exposure to infection increases the risk of HE and mortality rates. Appropriate prevention of infection and the use of antibiotics for cirrhotic patients at risk for HE are needed.

## Introduction

Hepatic encephalopathy (HE) is a commonly encountered complication in cirrhotic patients with advanced liver disease or portosystemic shunts. The incidence of HE ranges from 2% to 20% per year in patients with liver cirrhosis.[1–3] HE is associated with increased morbidity and mortality as well as significant utilization of health care resources.[4–6] Identifying risk factors for HE would be paramount for implementing preventive measures to improve overall outcomes for cirrhotic patients. When HE is diagnosed, underlying precipitating factors should be sought and treated first. Common culprits include gastrointestinal bleeding, infection, constipation, excessive dietary protein, hypovolemia, shock, hypokalemia, alkalosis, surgical portosystemic shunts or transjugular intrahepatic portosystemic shunts, hyponatremia, and medications such as opiates and benzodiazepines.[7,8]

HE is a reversible neuropsychiatric condition, and elevated ammonia level in the serum has been considered the primary pathophysiologic cause. It is widely accepted that ammonia is derived primarily from enteric bacterial flora.[7] Bacterial infection is present at admission (community-acquired infections) or develops during hospitalization in patients with liver cirrhosis (nosocomial and health care–related infections), and it occurs in more than 50% of hospitalized cirrhotic patients.[9] Spontaneous bacterial peritonitis (SBP), urinary tract infections (UTI), pneumonia, and cellulitis are the most frequent infections among cirrhotic patients in different settings.[7,9] Furthermore, ammonia toxicity is greatly attributed to fecal bacteria. *Helicobacter pylori* (*H*. *pylori*) in the stomach hydrolyzes urea and converts it to ammonia, which can be rapidly absorbed and increases blood ammonia concentrations in *H*. *pylori–*infected patients with cirrhosis.[10–12] However, ammonia levels are not related to severity of HE.[13] Whether eradication therapy for *H*. *pylori–*infected patients can lower ammonia levels and the risk of HE development remains inconclusive.[14]

Infections in cirrhotic patients are detrimental to hepatic functions and increase mortality fourfold.[15–17] To date, there is not enough empirical evidence of risk factors for HE in cirrhotic patients to support a prevention strategy for HE. To determine the independent effect of infections on the development of HE, we used a nested case-control design to avoid incorrect sampling of cases and controls and compared individual infections according to system and intensity.

## Materials and methods

The study was reviewed and approved by the Institutional Review Board and Ethics Committee of Chang Gung Medical Foundation in Taoyuan, Taiwan (IRB #201601548B1). All personal identifying information for patients was anonymized; therefore, the need for informed consent was waived for the study.

### Data sources

Data were extracted from the Longitudinal Health Insurance Database 2000 (LHID 2000) of 1 million individuals who were randomly sampled from the year 2000 Registry for Beneficences of 23.75 million individuals involved in Taiwan’s National Health Insurance (NHI) program.[18] The Taiwan NHI is a single-payer health insurance program that covers 99.9% of Taiwan’s population.[19] The LHID 2000 contains demographic information, diagnostics, medical treatments, prescriptions, and total costs from January 1, 1997 to December 31, 2012. Data analysts were staff members of the Center for Medical Informatics and Statistics at Kaohsiung Medical University, a site of the Application of Health and Welfare Informatics of the Ministry of Health and Welfare in Taiwan.

### Study design and population

**Fig 1:**
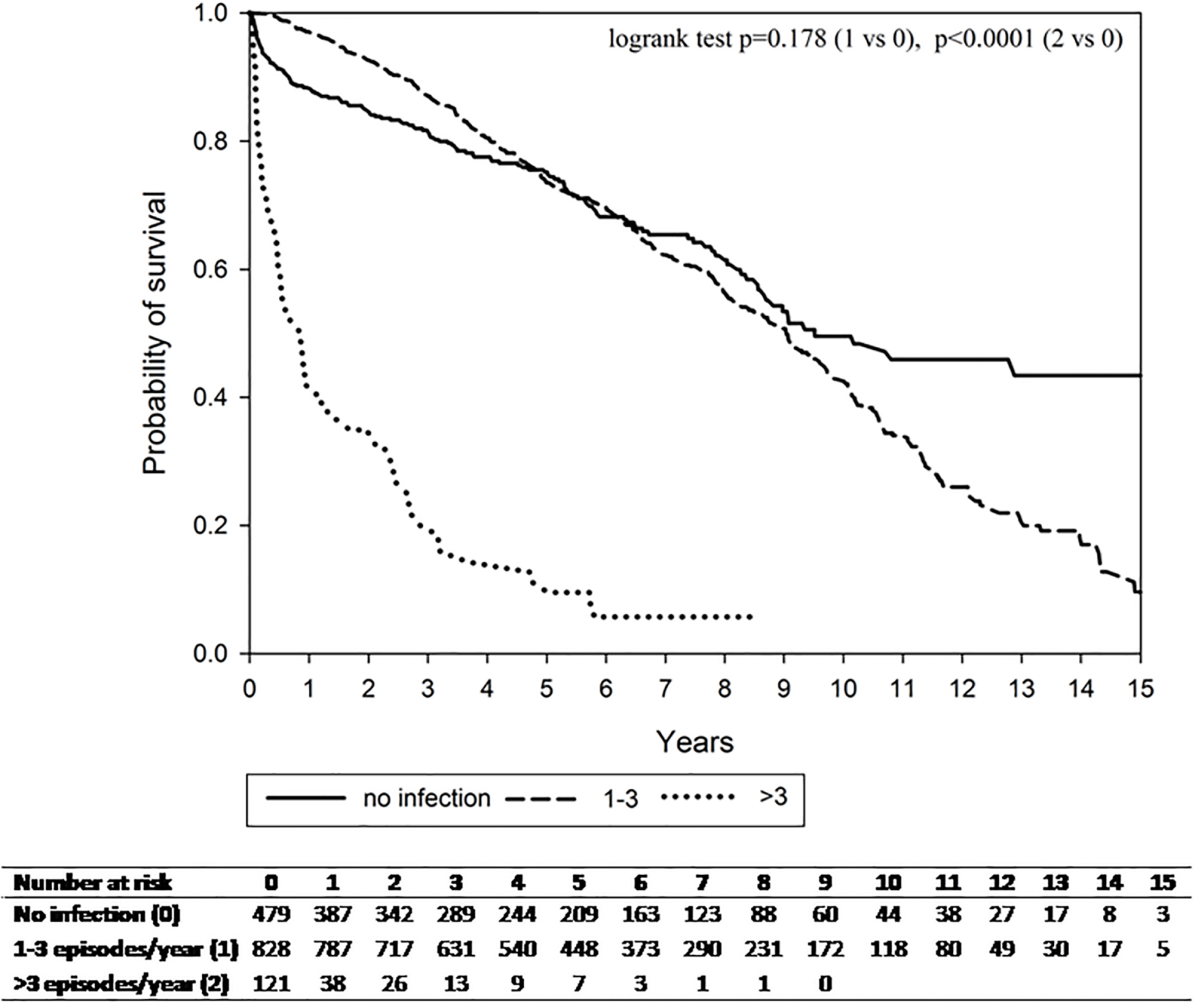
All-cause mortality among cirrhotic patients by frequency of infection episodes.

The population-based case study involved a cohort of patients aged older than 18 years with a hospital discharge diagnosis of liver cirrhosis (LC) (International Classification of Diseases, 9^th^ edition [ICD-9], codes 571.5, 571.2, 571.6) recorded in the LHID 2000 between 1997 and 2012 (Fig 1). The study index date was defined as the date of the first ICD-9 code for LC was assigned. Patients were excluded if they had prior peptic ulcer disease (ICD-9 531, 532, 533, 534), malignancy (140–239), HE (572.2), ever underwent a transjugular intrahepatic portosystemic shunt procedure (33113A, 3313B), or had infections including antimicrobial combination therapy or a diagnosis of *H*. *pylori* (041.86), pneumonia (481–487), spontaneous bacterial peritonitis (SBP) (567.2, 567.8, 567.9), sepsis (038, 020.0, 790.7, 112.81), UTI (590.1, 595.0, 595.9, 599.0), biliary tract infection (574.00, 574.01, 574.1, 574.30, 574.31, 574.4, 574.60, 574.61, 574.80, 574.81, 576.1, 575.0), cellulitis (681, 682, 728.86), inflammatory disease of the central nervous system (324, 320), septic arthritis (711), endocarditis (421), perianal abscess (566), or liver abscess (572.0) recorded as both inpatient and outpatient claims within 365 days before the index date. *H*. *pylori* infection and eradication triple or quadruple therapy was defined as proton-pump inhibitor (PPI) or histamine type 2 receptor antagonists (H2RA) plus clarithromycin or metronidazole plus amoxicillin or tetracycline, with or without bismuth for 7–14 days.[20,21]

All patients were followed from the first date of LC until death, withdrawal from the NHI program, or the last date used for the dataset (December 31, 2012). Patients in the case group who developed overt HE with more severe symptoms (Grade III-IV) requiring hospital admission for treatment during the follow-up period were defined by an ICD-9 code (572.2) for HE at hospital discharge.[22,23] For controls, the LC group without HE during follow-up was identified and subsequently matched to each HE case. The 1:1 exact matching for age at the index date within a 2-year difference, sex, and propensity score (including age at the index date, sex, Charlson comorbidity index [CCI] score, year of the index date, and length of follow-up in months) was performed for cases and controls.[24]

### Covariates

Patients’ demographic information, time at HE diagnosis, length of follow-up, comorbidities, liver diseases, and liver transplantation (LT) (ICD9, V427) were identified within 365 days prior to the index date. Comorbidities were assessed by the CCI using ICD-9 codes with at least two records from inpatient, emergency, and outpatient claims. Prevalent liver diseases were hepatitis B virus (HBV) infection (070.2, 070.3, V0261), hepatitis C virus (HCV) infection (070.41, 070.44, 070.51, 070.54, 070.70, 070.71, V0262), other viral hepatitis (V0269), alcohol-related liver disease (ALD) (571.0–571.3), decompensated cirrhosis with esophageal variceal bleeding (456.0, 456.20, 530.82), ascites (789.5, 567.2,567.8, 567.9, ICD-9 procedure 54.91), and others such as jaundice (782.4), portal hypertension (572.3), hepatorenal syndrome (572.4), other sequelae of chronic liver disease (572.8), and hepatic cellular carcinoma (HCC) (1550) within 1 year before the index date.

During follow-up, ICD-9 codes for infections recorded on inpatient claims were obtained. To investigate the dose relationship between infection and HE risk, the number of infections during hospitalization was categorized as 0, 1–3, and more than 3. Undergoing endoscopy for upper gastroenterology bleeding (billing code 47043B with the exclusion of ICD-9 44.43) and developing HCC and decompensated cirrhosis were accounted for by all-cause mortality analysis.

### Statistical analysis

Descriptive statistics and counts with proportions were presented as the mean (standard deviation [SD]) for categorical data and as the median (25^th^-75^th^ percentile) for continuous data. Categorical variables were compared between groups using the χ^2^ test. Multiple logistic regression models were used to calculate adjusted odds ratios (aOR) with 95% confidence intervals (CI) for development of HE among cirrhotic patients. Model 1 included exposure to any infection, model 2 included frequency of infection, and model 3 included individual infections. All three models included patient demographic characteristics, CCI scores, *H*. *pylori* therapy initiation, HCC, and decompensated cirrhosis development that may predispose to HE. The time-dependent Cox proportional hazards regression model was used to calculate the adjusted hazard ratio (aHR) with 95% CI for all-cause mortality and accounted for potential time-varying covariate effects.[25] The independent effect of HE in the model was adjusted according to infection, patient demographic characteristics, CCI score, *H*. *pylori* therapy, HCC, and decompensated cirrhosis development. The level of statistical significance was 5%, and a two-sided *P*<0.05 was considered significant. All statistical analyses were performed using SAS software version 9.4 (SAS Institute Inc., Cary, NC).

## Results

### Baseline patient characteristics

In the study cohort, 6078 cirrhotic patients (42%) who ever had an infection before development of HE were excluded (S1 Fig). The incidence of HE among patients with liver cirrhosis was 11.3% (2136 of 18824 cirrhotic patients). The demographic characteristics of the LC patients with and without HE are shown in S1 Table. Using the predefined matching criteria, 714 patients with HE during hospitalization (cases) and 714 randomly selected patients without HE (controls) were analyzed. In the LC-matched cohort, the mean age was 55.74±13.35 years and 548 (76.75%) patients were male. The demographic characteristics of the LC-matched cohort are shown in Table 1.

**Table 1:** Baseline characteristics of cirrhotic patient cohort.

Characteristics	Case group (n = 714)		Control group (n = 714)		P value
	N	%	N	%	
**Age, years (mean ± SD) at LC diagnosis**	55.74±13.35		55.74±13.35		1.000
≤ 49	247	34.59%	247	34.59%	1.000
50–59	194	27.17%	194	27.17%	
60–69	140	19.61%	140	19.61%	
≥70	133	18.63%	133	18.63%	
**Gender**					
Male	548	76.75%	548	76.75%	1.000
Female	166	23.25%	166	23.25%	
**CCI score (mean ± SD)**	1.39±1.22		1.40±1.25		0.813
**Charlson comorbid Index**					
Acute myocardial infarction	6	0.84%	2	0.28%	0.288
Congestive heart failure	32	4.48%	39	5.46%	0.394
Peripheral vascular disease	8	1.12%	14	1.96%	0.197
Cerebral vascular accident	36	5.04%	51	7.14%	0.097
Dementia	10	1.40%	10	1.40%	1.000
Pulmonary disease	75	10.50%	96	13.45%	0.087
Connective tissue disorder	9	1.26%	6	0.84%	0.436
Liver disease	458	64.15%	426	59.66%	0.081
Diabetes	172	24.09%	176	24.65%	0.805
Diabetes complications	34	4.76%	40	5.60%	0.474
Paraplegia	3	0.42%	6	0.84%	0.506
Renal disease	44	6.16%	35	4.90%	0.298
Severe liver disease	3	0.42%	2	0.28%	1.000
HIV	2	0.28%	2	0.28%	1.000
**Liver-related diagnosis**					
Alcoholism	161	22.55%	125	17.51%	0.017
Viral hepatitis	143	20.03%	138	19.33%	0.739
HCC	0	0.00%	0	0.00%	--
Decompensated cirrhosis	77	10.78%	52	7.28%	0.021
**Use of PPI**	32	4.48%	41	5.74%	0.28

LC, liver cirrhosis; CCI, Charlson comorbid index; SD, standard deviation; HCC, hepatic cellular carcinoma; PPI = proton pump inhibitors,

Case: patients with HE, control: patient without HE, Patient’s comorbid conditions were identified within 1 year prior to the date of LC diagnosis

### Ascertainment of risk factors

The potential risk factors for HE were categorized from the study index date to the first event of HE during hospitalization. As shown in Table 2, a higher proportion of patients in the case group (58.82%) than in the control group (30.81%) was exposed to any type of infection. *H*. *pylori* infection (13.31% vs. 8.68%; p<0.01), pneumonia (14.99% vs. 10.50%; p = 0.01), spontaneous bacterial peritonitis (SBP) or peritonitis (14.29% vs. 2.52%; p≤0.001), sepsis (25.63% vs. 9.52%; p<0.001), UTI (18.77% vs. 11.20%; p<0.0001), biliary tract infection (7.14% vs. 3.22%; p<0.001), and cellulitis (11.62% vs. 3.98%; p = 0.02) increased the risk of HE. Hospitalization due to infection occurred for 6.44±42.26 of the case group and 0.87±1.27 of the control group.

**Table 2:** Exposure of risk factors for hepatic encephalopathy.

Outcomes	Case group (n = 714)		Control group (n = 714)		P value
	N	%	N	%	
**Infections**	420	58.82%	220	30.81%	<.0001
H Pylori infection or therapy	95	13.31%	62	8.68%	0.005
Pneumonia	107	14.99%	75	10.50%	0.011
SBP and unspecified peritonitis	102	14.29%	18	2.52%	<.0001
Sepsis without infection focus	183	25.63%	68	9.52%	<.0001
Urinary tract infection	134	18.77%	80	11.20%	<.0001
Biliary tract infection	51	7.14%	23	3.22%	0.001
Cellulitis	83	11.62%	57	7.98%	0.021
Central nerve system infection	3	0.42%	4	0.56%	1.000
Septic arthritis	9	1.26%	3	0.42%	0.08
Infective endocarditis	0	0.00%	0	0.00%	--
Perianal abscess	6	0.84%	7	0.98%	0.781
Liver abscess	9	1.26%	7	0.98%	0.615
**Numbers of infectious hospitalization** (n = 420)					
mean ± SD	6.44±42.26		0.87±1.27		0.007
No infection	294	41.18%	494	69.19%	<.0001
**H Pylori therapy initiated after LC**	69	9.66%	44	6.16%	0.014
≤ 3 months	39	5.46%	20	2.80%	0.251
3–12 months	15	2.10%	12	1.68%	0.501
>12 months	15	2.10%	12	1.68%	0.501
**PUB with endoscopy therapy**	9	1.26%	12	1.68%	0.51
**Decompensated cirrhosis**					
EV Bleeding	5	0.70%	4	0.56%	1.000
Ascites	10	1.40%	3	0.42%	0.051
others	1	0.14%	0	0.00%	1.000

SBP, spontaneous bacterial peritonitis; LC, liver cirrhosis; SD, standard deviation; PUB, peptic ulcer bleeding, EV, esophageal varices;

The exposure of risk factors for hepatic encephalopathy was categorized from the date of LC diagnosis to the first event of hospitalized HE.

### Risk factors associated with hepatic encephalopathy

The proportion of the case group ever exposed to infection was higher than that of the control group (69.19% vs. 41.18%; p<0.001). Tables 3 and 4 show risk factors associated with HE. Infections were associated with hepatic encephalopathy development (aOR, 3.04; 95% CI, 2.44–3.78; p<0.0001). Compared to patients without any infection, patients with 1 to 3 episodes of any type of infection had approximately threefold odds of developing HE (aOR, 2.68; 95% CI, 2.13–3.37; p<0.001), and the odds increased for patients with more frequent infections (aOR, 10.27; 95% CI, 5.17–20.4; p<0.001) when controlling for age at LC diagnosis, gender, CCI score, and severity of liver diseases.

**Table 3:** Risk factors associated with hepatic encephalopathy.

	Model 1			Model 2		
	OR	95%CI	P value	OR	95%CI	P value
**Any infection**	3.04	2.44–3.78	0.0001			
**Number of infection per year**						
0 to<3				2.68	2.13–3.37	<.0001
≥3				10.27	5.17–20.4	<.0001
**Age at LC diagnosis, years**						
50–59 vs ≤ 49	0.97	0.73–1.28	0.821	0.96	0.72–1.27	0.757
60–69 vs ≤ 49	0.89	0.64–1.24	0.502	0.90	0.65–1.25	0.515
≥ 70 vs ≤ 49	0.92	0.65–1.29	0.61	0.86	0.61–1.22	0.401
Male vs Female	1.05	0.79–1.4	0.733	1.02	0.77–1.37	0.871
**CCI score**	0.98	0.89–1.07	0.637	0.97	0.89–1.07	0.553
**Prior to HE development**						
*H*. *Pylori* therapy	1.29	0.86–1.95	0.225	1.35	0.90–2.04	0.153
HCC	3.64	0.58–22.86	0.169	3.80	0.61–23.52	0.152
PUB	0.43	0.16–1.15	0.093	0.45	0.17–1.22	0.115
Decompensated cirrhosis	2.29	0.77–6.86	0.138	2.09	0.70–6.29	0.19

LC, liver cirrhosis; CCI, Charlson comorbid index; OR, odds ratio; CI, confidence interval; HCC, hepatic cellular carcinoma; PUB, peptic ulcer bleeding;

**Table 4:** Individual type of infection associated with hepatic encephalopathy.

	Model 3		
	OR	95% CI	p
*H*. *Pylori* therapy	1.34	0.88–2.04	0.178
Pneumonia	0.96	0.67–1.36	0.809
SBP and unspecified peritonitis	5.13	3.03–8.69	<.0001
Sepsis without infection focus	2.54	1.82–3.53	<.0001
Urinary tract infection	1.38	0.99–1.94	0.061
Biliary tract infection	2.03	1.20–3.46	0.009
Cellulitis	1.13	0.77–1.66	0.543
Perianal abscess	0.81	0.25–2.58	0.722
Liver abscess	0.70	0.24–2.07	0.523
Age at LC, years			
50–59 vs ≤ 49	0.94	0.71–1.24	0.653
60–69 vs ≤ 49	0.96	0.69–1.34	0.819
≥ 70 vs ≤ 49	1.00	0.71–1.42	0.983
Male vs Female	1.15	0.86–1.54	0.353
CCI score	1.00	0.91–1.09	0.957
Prior to HE development			
HCC	2.93	0.46–18.7	0.255
PUB	0.45	0.16–1.26	0.126
Decompensated cirrhosis	2.19	0.69–6.92	0.182

OR, odds ratio; CI, confidence interval; SBP, spontaneous bacterial peritonitis; CCI, Charlson comorbid index; HCC, hepatic cellular carcinoma; PUB, peptic ulcer bleeding;

Central nerve system infection and septic arthritis were excluded from the model 3 due to number of patient <5 in case or control group.

In model 3 (Table 4), an independent effect associated with HE development was observed among patients who ever had SBP (aOR, 5.13; 95% CI, 3.03–8.69; p<0.0001), sepsis (aOR, 2.54; 95% CI, 1.82–3.53; p<0.0001), or biliary tract infection (aOR, 2.03; 95% CI, 1.20–3.46; p = 0.009). Although UTI was associated with an additional 38% risk for HE, its effect did not reach statistical significance in this study cohort.

### Risk factors associated with all-cause mortality

The overall mortality rate was 43.1% (n = 615) for the matched study cohort and 76% for those who ever had an infection before HE development. The overall survival rate was not statistically different between patients with and without development of HE (48.6% vs. 37.54%; P = 0.081) (S2 Fig)

Table 5 shows risk factors associated with all-cause mortality in the time-dependent Cox model. Mortality risks significantly increased with an infection frequency >3 (aHR, 8.09; 95% CI, 6.01–10.87; p<0.0001), age 60–69 years (aHR, 1.52; 95% CI, 1.19–1.95; p = 0.001), age ≥70 (aHR, 2.49; 95% CI, 1.95–3.18; p<0.0001), higher CCI score (aHR, 1.11; 95% CI, 1.04–1.18; p = 0.001), and worse liver diseases (aHR, 1.71; 95% CI, 1.08–2.72; p = 0.023). However, *H*. *pylori* therapy during follow-up had negative effects on mortality (aHR, 0.74; 95% CI, 0.58–0.96; p = 0.023). Fi 1 shows that the survival rate was lower for patients with frequent infections.

**Table 5:** Risk factors associated with all-cause mortality.

Characteristics	HR	95%CI		P value
Patients with HE	1.03	0.87	1.22	0.711
Frequency of infection				
1–3 vs 0	1.04	0.86	1.27	0.681
≥ 3 vs 0	8.09	6.01	10.87	<.0001
Age, years				
50–59 vs ≤ 49	1.23	0.99	1.54	0.064
60–69 vs ≤ 49	1.52	1.19	1.95	0.001
≥ 70 vs ≤ 49	2.49	1.95	3.18	<.0001
Gender				
Male vs Female	1.20	0.99	1.46	0.065
CCI score	1.11	1.04	1.18	0.001
During entire follow-up				
*H Pylori* therapy	0.74	0.58	0.96	0.023
HCC	0.98	0.50	1.95	0.961
PUB	0.59	0.33	1.06	0.077
Decompensated cirrhosis	1.71	1.08	2.72	0.023

HE, hepatic encephalopathy; LC, liver cirrhosis; CCI, Charlson comorbid index; HCC, hepatic cellular carcinoma; PUB, peptic ulcer bleeding; HR, hazard ratio;

Survival outcome was determined from the date of LC diagnosis to the death event, withdrawn or the latest date in the dataset (December 31, 2012), whichever came first.

## Discussion

HE is one of the most debilitating complications of cirrhosis because it greatly affects society and individual patients and their caregivers.[5,6] Bacterial infections are common in patients with LC; however, the epidemiological features of infection associated with HE have not been clearly demonstrated. In the present study, we found the following. First, infection was an independent risk for HE, requiring hospitalization among cirrhotic patients. Second, more frequent infections significantly increased the dose-response risk of HE development and mortality. In addition, prevalent infections such as SBP and unspecified peritonitis were strong risk predictors of HE among cirrhotic patients.

Intestinal bacterial overgrowth is associated with higher ammonia production and higher serum ammonia concentrations, resulting in the induction of encephalopathy through the promotion of cerebral edema, modulation of the blood–brain barrier, and neuro-inhibition.[26] The present population-based epidemiological study confirmed that prior to the development of HE, cirrhotic patients with HE were more likely to have been exposed to infection than those without HE (58.81 vs. 30.81%; p<0.0001). The significant predictive factors of HE development for cirrhotic patients were SBP and unspecified peritonitis, sepsis, and biliary tract infection. UTI (30%) and pneumonia (25%) were the common sites of infection among cirrhotic patients, which is consistent with previous cirrhotic cohorts.[7] However, the individual predictive value of HE was not confirmed in the present study cohort. When the intensity of exposure to infection associated with HE development was examined, our data showed that a subgroup of cirrhotic patients with ≥3 infectious episodes per year is at higher risk for HE than those with fewer exposures.

More frequent exposure to infection (≥3 episodes per year) was a strong predictive factor for all-cause mortality in cirrhotic patients. The adjusted morality (aHR, 1.03; 95% CI, 0.87–1.22; p = 0.711) revealed no statistical difference between patients with and without HE. Although the therapeutic effectiveness of cirrhotic encephalopathy needs further investigations, these findings suggest that HE occurrence was not independent of survival outcome. In the present study, effect of HE occurrence on overall mortality among cirrhotic patients was assessed in time-dependent Cox model to justify cirrhosis complications that occurred before end of the study period. Future applications in survival research, it is important to consider the presence of competing events during follow-up, i.e. receiving liver transplantation, death on first HE event that might influence the results and interpretations in conventional Cox proportional hazard model.

As previously reported, a history of infection in the past 12 months, advanced liver disease (model of end-stage liver disease score ≥15), and diagnosis of malnutrition were independent predictors of infection and sepsis.[9] The present study results support that routine screening and identifying who is at risk for bacterial infections among hospitalized cirrhotic patients would facilitate compliance with empirical antibiotic therapy and may prevent resistance and worsening infection.[7]

The results of the present study support that *H*. *pylori* is more prevalent in cirrhotic patients with HE than in those without HE (13.31% vs. 8.68%; p = 0.005); however, the administration of standard combinations of *H*. *pylori* eradication regimens (22%) did not benefit HE prevention (aOR, 1.34; 95% CI, 0.88–2.04; p = 0.178) after adjusting for other sites of infection and deterioration of liver cirrhosis. It is worth noting that eradication of *H*. *pylori* was significantly associated with a 28% lower risk of mortality for patients with liver cirrhosis. Similarly, Dasani et al observed that symptoms in infected encephalopathic patients improved following *H*. *pylori* eradication therapy.[27]

Based on these study results and previous evidence,[14] the beneficial effects of eradication therapy for *H*. *pylori* are insufficient for recommending the use of this therapy in clinical practice. Because there are geographic differences in the incidence and prevalence of *H*. *pylori* infection and antimicrobial resistance and in the availability of medications and endoscopy, cirrhotic patients with a history of peptic ulcer, gastric mucosa–associated lymphoid tissue lymphoma, or history of endoscopic resection of early gastric cancer should be tested for *H*. *pylori* infection and administered the most appropriate combination of antibiotic regimens recommended in the clinical setting.[28,29]

Bajaj et al report the recent results of an open-label, randomized, standard of care (SOC) controlled study using fecal microbiota transplantation (FMT) in patients with recurrent HE. Twenty patients received a single FMT enema in combination with SOC and a 5-day antibiotic pre-treatment or SOC-only were followed for 150 days. During the follow-up, none of the HE episodes developed in the FMT group, compared with 5 (50%) in the SOC-only group.[30] The FMT group had cognitive function improvement and a lower rate of serious adverse event (20% vs 80%) than the SOC-only group. [30] Although FMT has shown promising results in the treatment of overt HE, stronger evidence on efficacy, long-term safety and which patients with HE should be considered are needed. A registry assessing short- and long-term patient outcomes after FMT has been developed in this regard. [31]

A systematic review with meta-analysis synthesized 21 trials, 1420 participants and suggest that probiotics has a beneficial effect on HE symptoms recovery (10 trials, 574 participants, RR 0.67, 95% CI 0.56–0.79) and development of overt HE (10 trials, 585 participants, RR 0.29, 95% CI 0.16–0.51) comparing to no intervention. [32] Serum ammonia level was lower (10 trials, 705 participants, mean difference -8.29 umol/L, 95% CI -13.7– -3.41) and slightly imporved quality of life for pariticipants with probiotcs. There were no reliable difference between probiotics and lactulose.[32] Considerable efforts of research in the field of gut microbiota are needed for the future therapeutic use in overt HE.

The overall mortality rate was high among cirrhotic patients with HE (48.6% of matched cases of HE and 51.26% of all HE cases) in the present study cohort, and 76% of them were exposed to infection before HE development. The present findings support other studies that have shown infections in cirrhotic patients with HE present additional risks of worsening outcomes.[33–35] For example, acquisition of infection during the mild stage of HE was significantly associated with progression to the advanced stage.[33] Moreover, recent reports have shown that bacterial infections were significantly associated with increased overall mortality among patients with HE.[34,35]

Most previous studies regarding the influence of the microbiota on LC were highly diverse in design and involved small numbers of patients in single-center settings.[14,36] One of the strengths of the present study was its relatively large sample size derived from a population-based cohort, thus allowing for the investigation of independent associations between individual sites of infection and HE development. The advantages of longitudinal data have enabled us to further investigate the dose-response association between infection and risk of HE development.

To date, the mechanism that could further induce HE development and deterioration of patient outcomes among those with LC remains unclear. Our data focusing on exposure to infection and the probability of the first episode of HE have relevant clinical implications for the detection and prevention of cirrhosis for high-risk patients. First, a synergistic effect of infections is likely to be important in developing HE. Prior exposure to infection and medication history is easy to obtain and should be identified during routine practice for those cirrhotic patients at higher risk for HE.

In addition, recommended empirical therapy for bacterial infections should be closely monitored and justified according to the prevalence of resistant pathogens.[7] Few prophylactic therapy strategies are recommended for patients with previous episodes of SBP and variceal bleeding.[37] Studies focusing on who will benefit from prevention of the first HE episode and what should be included in the standard of care therapy are needed. Despite the diverse range of bacterial pathogens, it has been suggested that interactions between gut microbiota, etiology of chronic LC, and host-related precipitating factors (e.g., malnutrition, low protein ascites <1.5 g/dL, and alcohol consumption)[38] should be incorporated into an algorithm for standard of care therapy.[8]

It is worthy to noting that acute kidney injury (AKI) is a recognized mortality predictor in cirrhotic patients with infection. The 30-day mortality was 10-fold higher in cirrhotic patients admitted with infection who developed AKI than those who did not. Advancing stages of AKI in cirrhotic patients were associated with a higher incidence of bacteremia, pneumonia and UTI, and cirrhosis-related encephalopathy and spontaneous bacterial peritonitis (SBP). [39]

For caring cirrhotic patients with infection, factors associated with worsening HE should be identified and closely monitored, including diuretic therapy and hypokalemia which may facilitate the conversion of ammonium (NH4) to ammonia (+NH3) and lead to acute mental dysfunction.[26] Furthermore, due to kidney involved both excretion and production of ammonia, early detection of hypovolemia and AKI, and prevention of patients affected by this disease (e.g., infection, diuretic therapy) are imperative to better survival outcome.[26,39]

This study was subject to certain limitations common to studies using claims data. First, laboratory results regarding the severity of LC and index HE are not available in the NHI dataset. Using the liver disease diagnosis as a proxy for the presence of decompensated cirrhosis (esophageal varices bleeding, ascites) at baseline and during years of follow-up can minimize the potential bias when estimating risk factors for the development of HE. In addition, there is a lack of bacterial culture results to determine microbial diversity and the effects of antimicrobial therapy for HE. We used the advanced stage HE diagnosis at hospital discharge, which could underestimate the incidence of mild infections without hospitalization; however, it ensured diagnostic infection specificity. Furthermore, other unmeasured or unknown health factors for HE development, including actual alcohol use, dietary protein intake, and medication use, among cirrhotic patients may result in a biased estimate of exposure to infection, thus limiting the generalizability of the current study results to different cirrhotic populations.

In conclusion, bacterial infections, especially SBP and unspecified peritonitis, sepsis, and biliary tract infection, are common and important predictors of the development of HE. Furthermore, a dose-response effect associated with an increased risk of HE highlights the need to promote appropriate infection prevention strategies and the use of antibiotic therapy for cirrhotic patients with different precipitating risks for HE. Additional research is needed regarding the associations between antibiotic therapy and the occurrence of HE or changes in its severity.

## Supporting information

S1 Fig Schematic flowchart of study design. (TIF)

S2 Fig All-cause mortality among cirrhotic patients by occurrence of hepatic encephalopathy. (TIF)

S1 Table Baseline characteristics of cirrhotic patient cohort before matching. (DOCX)

## Abbreviations

CI: Confidence interval
*H. pylori*: *Helicobacter pylori*
HE: Hepatic encephalopathy
ICD-9-CM: International Classification of Diseases, Ninth Revision, Clinical Modification
LC: liver cirrhosis
LHID 2000: Longitudinal Health Insurance Database 2000
NHI: National Health Insurance
OR: Odds ratio
PPI: Proton pump inhibitor
SBP: Spontaneous bacterial peritonitis
UTI: urinary tract infections
