# Supplementary figures and images for: Multiple bacterial infections increase the risk of hepatic encephalopathy in patients with cirrhosis
Source: PLoS One. 2018 May 10;13(5):e0197127. doi: 10.1371/journal.pone.0197127 (PMC5945008; doi:10.1371/journal.pone.0197127)

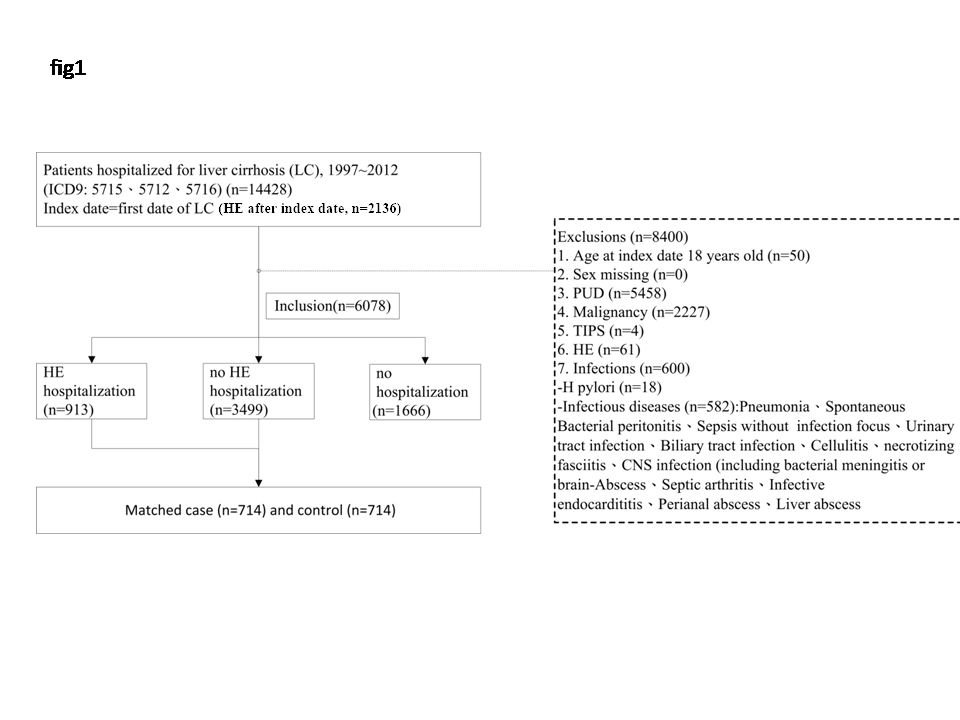

Supplement: S1 Fig — (TIF) [file pone.0197127.s001.tif]

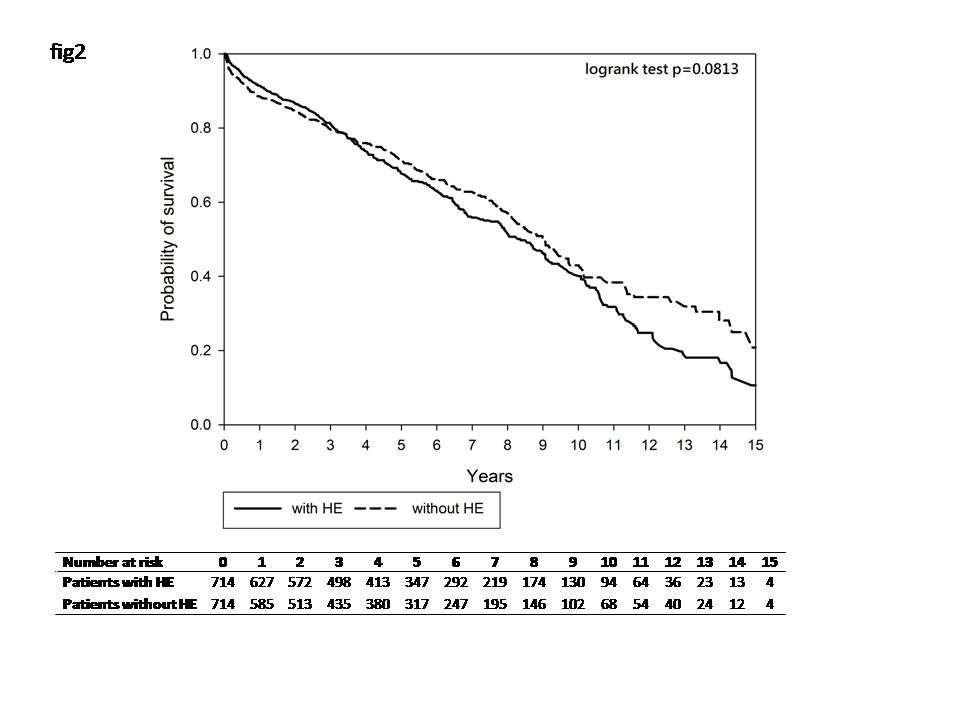

Supplement: S2 Fig — (TIF) [file pone.0197127.s002.TIF]
