# Supplementary material for: Multiple bacterial infections increase the risk of hepatic encephalopathy in patients with cirrhosis
Source: PLoS One. 2018 May 10;13(5):e0197127. doi: 10.1371/journal.pone.0197127 (PMC5945008; doi:10.1371/journal.pone.0197127)
Supplement: S1 Table — (DOCX) [file pone.0197127.s003.docx]

S1 Table. Baseline characteristics of cirrhotic patient cohort before matching

| **Characteristics** | **Patients with HE (n=913)** | |  | **Patients without HE (n=3499)** | |  | **p-value** |
| --- | --- | --- | --- | --- | --- | --- | --- |
|  | **N** | **%** |  | **N** | **%** |  |  |
| **Age, years (mean ± SD) at LC diagnosis** | 55.95± 13.74 | |  | 56.95± 14.74 | |  | 0.055 |
| ≤ 49 | 312 | 34.17% |  | 1174 | 33.55% |  | 0.045 |
| 50-59 | 241 | 26.40% |  | 793 | 22.66% |  |  |
| 60-69 | 183 | 20.04% |  | 747 | 21.35% |  |  |
| ≥70 | 177 | 19.39% |  | 785 | 22.43% |  |  |
| **Gender** |  |  |  |  |  |  |  |
| Male | 651 | 71.30% |  | 2457 | 70.22% |  | 0.523 |
| Female | 262 | 28.70% |  | 1042 | 29.78% |  |  |
| **Charlson comorbidity index (CCI) score (mean ± SD)** | 1.38±1.22 | |  | 1.42±1.25 | |  | 0.360 |
| **CCI** |  |  |  |  |  |  |  |
| Acute myocardial infarction | 7 | 0.77% |  | 25 | 0.71% |  | 0.869 |
| Congestive heart failure | 43 | 4.71% |  | 185 | 5.29% |  | 0.483 |
| Peripheral vascular disease | 12 | 1.31% |  | 48 | 1.37% |  | 0.894 |
| Cerebral vascular accidents | 47 | 5.15% |  | 272 | 7.77% |  | 0.006 |
| Dementia | 12 | 1.31% |  | 52 | 1.49% |  | 0.699 |
| Pulmonary disease | 99 | 10.84% |  | 525 | 15.00% |  | 0.001 |
| Connective tissue disorder | 11 | 1.20% |  | 46 | 1.31% |  | 0.794 |
| Peptic ulcer | 0 |  |  | 0 |  |  |  |
| Liver disease | 581 | 63.64% |  | 2167 | 61.93% |  | 0.344 |
| Diabetes | 210 | 23.00% |  | 739 | 21.12% |  | 0.218 |
| Diabetes complications | 43 | 4.71% |  | 188 | 5.37% |  | 0.423 |
| Paraplegia | 5 | 0.55% |  | 29 | 0.83% |  | 0.387 |
| Renal disease | 58 | 6.35% |  | 211 | 6.03% |  | 0.717 |
| Cancer | 0 |  |  | 0 |  |  |  |
| Metastatic cancer | 0 |  |  | 0 |  |  |  |
| Severe liver disease | 4 | 0.44% |  | 12 | 0.34% |  | 0.756 |
| HIV | 2 | 0.22% |  | 3 | 0.09% |  | 0.277 |
| **Other comorbid conditions** |  |  |  |  |  |  |  |
| Alcoholism | 193 | 21.14% |  | 486 | 13.89% |  | <.0001 |
| Viral hepatitis | 171 | 18.73% |  | 650 | 18.58% |  | 0.916 |
| HCC | 0 |  |  | 0 |  |  |  |
| Decompensated cirrhosis | 101 | 11.06% |  | 180 | 5.14% |  | <.0001 |
| PPI use | 38 | 4.16% |  | 159 | 17.42% |  | 0.619 |

HE, hepatic encephalopathy; LC, liver cirrhosis; HP, *Helicobacter pylori*; HIV, human immunodeficiency virus; HCC, hepatocellular carcinoma; PPI, proton pump inhibitor;
